# Disrupted brain networks underlying high‐fidelity memory retrieval in subjective cognitive decline: A task‐based fMRI study

**DOI:** 10.1002/alz.14431

**Published:** 2024-12-28

**Authors:** Wei Tang, Qinghe Zeng, Kaiqi Xie, Xiaoyu Cui, Xinhu Jin, Ying Han, Juan Li

**Affiliations:** ^1^ Center on Aging Psychology, CAS Key Laboratory of Mental Health, Institute of Psychology Chinese Academy of Sciences Beijing China; ^2^ Department of Psychology University of Chinese Academy of Sciences Beijing China; ^3^ Department of Neurology Xuanwu Hospital of Capital Medical University Beijing China; ^4^ State Key Laboratory of Digital Medical Engineering, Key Laboratory of Biomedical Engineering of Hainan Province, School of Biomedical Engineering Hainan University Sanya Hainan China; ^5^ Institute of Biomedical Engineering Shenzhen Bay Laboratory Shenzhen China; ^6^ Center of Alzheimer's Disease Beijing Institute for Brain Disorders Beijing China; ^7^ National Clinical Research Center for Geriatric Diseases Beijing China; ^8^ The Central Hospital of Karamay Xinjiang China

**Keywords:** generalized psychophysiological interaction, high‐fidelity retrieval, information transmission, integrating region, mnemonic similarity task, subjective cognitive decline, task fMRI

## Abstract

**INTRODUCTION:**

Subjective cognitive decline (SCD) is linked to memory complaints and disruptions in certain brain regions identified by molecular imaging and resting‐state functional magnetic resonance imaging studies. However, it remains unclear how these regions interact to contribute to both subjective and potential objective memory issues in SCD.

**METHODS:**

To address this gap, task‐based imaging studies are essential. The Mnemonic Similarity Task assessed high‐fidelity retrieval meanwhile MRI data measured group differences in activation and functional connectivity (FC) (calculated by generalized psychophysiological Interaction) between SCD individuals and normal controls.

**RESULTS:**

Worse high‐fidelity retrieval in SCD was associated with hypoactivation in the hippocampus, hyperactivation in the control network (CN), and reduced FC between the hippocampus and CN. The angular gyrus (AG) partially drives this disconnection.

**DISCUSSION:**

This study confirms objective cognitive deficits in SCD and highlights the AG's failure to integrate, addressing a gap in the literature that has primarily focused on the hippocampus and CN.

**Highlights:**

- Objective high‐fidelity retrieval deficits detected in older adults with SCD.
- Dysfunctional neural activations within retrieval networks impair memory accuracy.
- Reduced task‐based FC drives high‐fidelity retrieval deficits.
- The SCD‐related disruption of integrative function partly explains such deficits.
- Future studies may benefit from inspections on obstruction in cognitive process.

## 1 BACKGROUND

Subjective cognitive decline (SCD) is considered a preclinical stage of cognitive impairment in which older individuals report memory complaints but still perform normally on standardized neuropsychological tests.^1^, ^2^, ^3^, ^4^, ^5^, ^6^, ^7^ Previous evidence has shown that individuals with SCD tend to have a higher risk of progressing to cognitive impairment or Alzheimer's disease (AD) in the future.^8^, ^9^, ^10^, ^11^, ^12^, ^13^, ^14^

Molecular imaging and resting‐state functional magnetic resonance imaging (fMRI) studies have identified issues in certain brain regions among the SCD population, including pathological deposits of amyloid‐beta (Aβ) and tau, as well as disruptions in resting‐state functional connectivity (FC). Research has identified typical AD‐related pathologies, such as Aβ and tau deposition in the hippocampus, medial prefrontal cortex (mPFC), precuneus, and posterior cingulate cortex (PCC) among individuals with SCD.^7^, ^15^, ^16^, ^17^, ^18^, ^19^, ^20^ Additionally, dysfunction in hippocampal activation and resting‐state FC between the hippocampus and cortical regions has been observed in various SCD cohorts.^4^, ^7^, ^9^, ^21^, ^22^, ^23^, ^24^, ^25^ These findings correlate with memory concerns,^24^ suggesting that damage in the hippocampus, frontal lobe, and precuneus may contribute to memory deficits, leading to subjective complaints in individuals with SCD.

However, a key question remains: is there objective memory impairment associated with SCD? The relationship between the identified abnormal brain regions and the subjective complaints of SCD, as well as potential objective memory deficits, is not yet clear. Specifically, it is uncertain how these brain regions interact to result in both subjective and potential objective memory issues in individuals with SCD. This ambiguity arises partly because neural activation during cognitive processes, such as memory encoding and retrieval, is not fully captured by resting‐state FC, which reflects neural activity in the absence of cognitive tasks.^24^ The association between alterations in the specific memory process and pathological deposition also remains unclarified.

To address this gap, task‐based imaging studies are essential. By observing the brains of individuals with SCD during task performance and comparing them with cognitively normal elderly individuals, we can connect SCD's task performance with underlying brain activity, thereby answering these questions.

Research on AD risk gene carriers has provided valuable insights.^26^ As a remarkably sensitive and ecological paradigm on memory retrieval,^21^, ^22^, ^23^, ^27^, ^28^ the MST requires subjects to precisely encode and retrieve the presented objects, which imitates the process of recognizing subtle differences among plenty of objects in everyday life. Results found AD risk gene carriers had difficulty in discriminating similar but not identical items, indicating that they could not retrieve objective and accurate information for the specific details of common visual items: high‐fidelity memory.^29^ The findings showed deficits in high‐fidelity retrieval even though they performed normally on neuropsychological tests. Moreover, AD risk gene carriers tended to recognize similar items as the same, demonstrating a preference for generalized recognition and retrieval without details (i.e., low‐fidelity memory).^29^ Utilizing high‐fidelity retrieval paradigms, studies have demonstrated objective memory declines at the early stages of AD,^21^, ^23^, ^26^ highlighting the potential of MST in identifying objective memory deficits associated with SCD.

The high‐fidelity retrieval is a complex cognitive process that involves the coordinated efforts of multiple brain regions^30^: hippocampus within medial temporal lobe (MTL) first extracts detailed memory representations. Then, the mPFC as well as other prefrontal regions within control network (CN) monitors conflicts between memory and inputs. Finally, precuneus region within CN would evaluate retrieval results for successful high‐fidelity retrieval.^31^, ^32^, ^33^ The regions associated with high‐fidelity retrieval closely align with those identified in imaging studies as problematic in SCD. Thus, the employment of MST in the task‐related fMRI would further elucidate how pathological alterations impact brain activities during retrieval processes.

Therefore, our research aims to employ high‐fidelity retrieval paradigms to (1) not only identify objective memory issues in SCD but, more importantly, (2) to directly link the previously identified dysfunctional brain regions with memory performance through task‐based fMRI. In other words, we seek to uncover the cognitive significance of the impaired brain regions and their functions identified in current research.

## 2 MATERIALS AND METHODS

### 2.1 Participants

A priori power analysis of the group main effect in repeated measures analysis of variance (ANOVA) has been conducted using G*Power 3.1. The analysis indicated that 56 participants would be sufficient to achieve 80% power with a medium to large effect size (*f* = 0.30) at a significance level of *α* = 0.05 and a correlation of 0.2 between conditions. A total of 210 community‐dwelling older adults in Beijing, China, were initially recruited and 66 participants were included after screening. SCD was diagnosed according to the criteria of Jessen and colleagues.^1^ The inclusion criteria for participants were: (a) over 60‐year‐old; (b) normal global cognition (Montreal Cognitive Assessment > 24)^34^; (c) education ≥ 9 years; (d) performed higher than 1.5 SD below the mean of norms in memory tests; (e) right‐handed; (f) no hearing, vision, or language deficit; (g) no contraindication for fMRI; (h) no mental or physical disease or neurological deficit that impairs cognitive function; (i) no anxiety (self‐rating anxiety scale ≤ 40)^35^ or depression (Center for Epidemiological Studies‐Depression ≤ 16)^36^ symptom. After the screening, in compliance with Subjective Cognitive Decline Questionnaire 9 (SCD‐Q9) plus criteria,^37^ older adults who scored >3 in SCD‐Q9 and simultaneously reported concerns about memory decline were assigned to the SCD group, while those who scored ≤ 3 were assigned to the NC group.

The study was approved by the Institutional Review Board of the Institute of Psychology, Chinese Academy of Sciences (H23104, protocol IPCAS2023014), and all participants completed written informed consents and were paid for their participation.

### 2.2 Materials

In each practice or experimental session, one set of 64 different pictures of everyday objects would be displayed to participants and the participants were expected to respond to these pictures. Such pictures could be attributed to foil items that would only be displayed once (16 pictures), pairs of old items (target items and their first‐time presentations, 16 pictures that were displayed twice), and pairs of similar items (16 pictures of lure items and 16 pictures of similar first‐time presentations). To avoid confusion, the “subsequent” items all referred to the first‐time presentations of target and lure items. Pictures in each session are completely different from those in other sessions. For each participant, seven sets (one for practice and six for experimental sessions) of 448 pictures would be displayed in total.

**RESEARCH IN CONTEXT**

1. **Systematic review**: Available research highlights associations between impairments in specific brain regions and memory complaints of individuals with subjective cognitive decline (SCD). However, it remains unclear whether SCD individuals exhibit objective memory decline and how the collaborations among these impaired regions affect specific memory processes.
2. **Interpretation**: SCD participants perform worse on the mnemonic similarity task (MST) compared to normal controls (NCs). This is linked to disruptions in the regions responsible for high‐fidelity retrieval, which is characterized by the hippocampal hypoactivation, hyperactivation in prefrontal regions within the control network (CN), and reduced hippocampal‐CN functional connectivity (FC) during high‐fidelity retrieval. Moreover, the failed integrative role of the angular gyrus (AG) partially explains the observed disconnection.
3. **Future directions**: Large cohort research is encouraged to validate the preliminary findings of this pioneer study. Future research should prioritize task‐based imaging techniques under cognitive neuroscience framework to better understand the specific cognitive processes affected by pathology.

### 2.3 Procedure and fMRI task

After all participants’ demographical information was collected, they were then asked to complete various neuropsychological tests of global cognition, episodic memory, verbal fluency, executive function, and emotion. The participants who matched the inclusion criteria were separated into SCD and NC groups according to scores of SCD‐Q9 and memory concerns. Subsequently, the imaging of each participant was collected using an event‐related design while they were completing an adapted version of MST.

**FIGURE 1:**
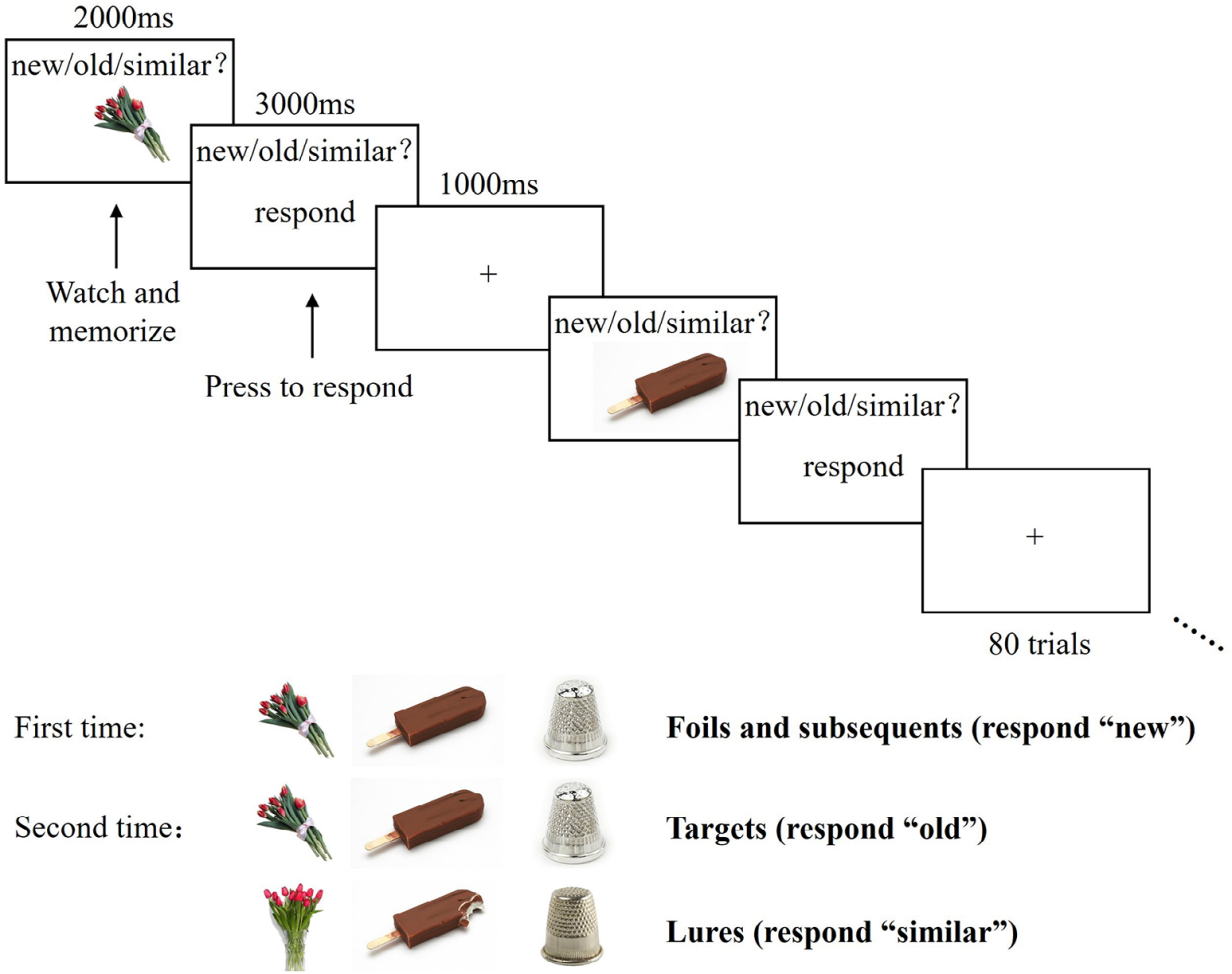
The adapted version of MST. For each trial, participants were expected to make “old”/“similar”/“new” judgments to lure items, target items, and foil along with subsequent items. First items include two categories of subsequent and foil items while second items include two categories of target and lure items. MST, mnemonic similarity task.

The adapted version of MST^27^, ^28^ consisted of one practice session and six experimental sessions. Each session included 80 trials that were presented in pseudorandom order, which could be first‐time presentations (foil and subsequent items) or second‐time presentation items (lure and target items). Notably, target and lure items would strictly be presented within 10–25 trials after the presentation of subsequent items. Each picture was presented for 2000 ms, during which the participants were asked to carefully indicate whether the object presented in the picture is “old and the same” (i.e., target), “similar but not identical” (i.e., lure) or “never seen before” (i.e., foil/subsequent) image^27^ and prepare to press the key. Later, an instruction of “please press the key to respond” would be presented for 3000 ms for the participants to respond “new/old/similar” correspondingly after the presentation of each item, followed by a 1000 ms inter‐stimulus‐interval reminding the participants to prepare for the next trial (detailed procedure refers to Figure 1). After a practice session, all participants would complete all six experimental sessions in a random order, and were expected to immediately respond, the response and reaction time were meanwhile collected.

### 2.4 MRI data acquisition

All imaging data were collected on a 3‐Tesla GE MRI scanner (GE discovery MR750, GE Healthcare, WI, USA) at the Magnetic Resonance Imaging Research Center, IPCAS. The functional MST task was collected using gradient EPI sequence: repetition time/echo time = 2000 ms/30 ms, flip angle = 90°, acquisition matrix = 64 × 64, slice thickness = 3.5 mm, voxel size = 3.5 × 3.5 × 3.5 mm^3^, 37 slices, 251 volumes (dummy scans = 5 volumes) with interleaved slice acquisitions. The structural MRI data were collected using Sagittal 3D BRAVO T1‐weighted sequence: repetition time/echo time = 7.4 ms/3.1 ms, flip angle = 12°, slice thickness = 1.0 mm, voxel size = 1.0 × 1.0 × 1.0 mm^3^, 192 slices.

### 2.5 fMRI data preprocessing

#### 2.5.1 Preprocessing of task fMRI

The fMRI data was preprocessed with statistical parametric mapping (SPM12). The preprocessing procedure includes slice‐time correction, realignment, co‐registration, segmentation and normalization to the Montreal Neurological Institute (MNI) template and smooth (6‐mm FWHM Gaussian Kernel). The first level (single subject) analyses were conducted using a full‐factorial general linear model (GLM) and six motion parameters were included as covariates. Only participants who showed acceptable head motion (maximal translation/rotation ≤ 3 mm/degree) were included. Among 66 recruited participants, 7 participants were excluded due to severe head motion when scanning fMRI, 3 participants were excluded due to misunderstanding the task, and 2 participants were excluded due to no concerns associated with SCD. The final sample consisted of 26 SCD participants (aged 61–72, *M*_age_ = 66.35, *SD*_age_ = 2.94, 10 males) and 28 NC participants (aged 61–71, *M*_age_ = 65.71, *SD*_age_ = 3.28, 9 males).

#### 2.5.2 Seed selection and generalized psychophysiological interactions

Seeds for generalized psychophysiological interactions (gPPI) were six regions of interest (ROIs) extracted from the regions of significant group effect shown in task fMRI analysis, which were left inferior frontal gyrus (L IFG), mPFC, anterior cingulate gyrus (ACC), right precuneus (R PreC), left middle frontal gyrus (L MFG) and left hippocampus. The preprocessing procedure and analysis were performed using CONN toolbox (v22a; www.nitrc.org/project/conn.)^38^ on SPM12. After default preprocessing including slice timing correction, realignment with correction of susceptibility distortion interactions, outlier detection, direct segmentation and MNI‐space normalization, smoothing with a 6‐mm full‐width half‐maximum (FWHM) Gaussian Kernel and simultaneous bandpass frequency filtering between 0.008 and 0.09 Hz (denoising), a gPPI model studying the changes in FC when responding “similar” to lure and responding “old” to lure were established in the first level analyses, while the other conditions were excluded from further analysis.

### 2.6 Data statistical analysis

#### 2.6.1 Behavioral data

IBM Statistical Package for Social Science (SPSS) version 26.0 (UNICOM, California, USA) was used for statistical analyses. Sex was compared with a chi‐squared test. Age, education, scores of neurological tests, and emotions were compared with T‐tests between the two groups. Proportions for responses (“new,” “old,” and “similar”) to each category of items (“first,” “target,” and “lure”) were recorded during the MST task. To study the group differences in memory accuracy, we conducted a 2 (group: NC vs. SCD) × 3 (item: target vs. lure vs. first) × 3 (response: old vs. similar vs. new) mixed ANOVA and focused on interaction effect. Of critical interest were the responses on the lure items between two groups, simple effect analyses were therefore performed to investigate the interaction effect of item × response in two separate groups. Furthermore, to control the response bias of responding “similar” to pictures never seen before, lure discrimination index (LDI), which represented the ability of precise retrieval after controlling response bias, was educated as the difference between the proportion of responding “similar” to lure item (correct hit) and the proportion of responding “similar” to new item (false alarm), LDI = [P(Similar|lure)‐P(Similar|First)].^21^, ^22^, ^26^ Correlations between LDI and imaging data were also conducted to assess the relationship between cognitive function and brain activities. In the present study, all *r* and *p*‐value pairs reflected Pearson correlation.

#### 2.6.2 Task fMRI

A total of 13 conditions were defined to model the different trials (see Table S1), while FN (responded “new” to foil items), LO (low‐fidelity retrieval, responded “old” to lure items) and LS (high‐fidelity retrieval, responded “similar” to lure items) were later analyzed, and FN served as an arbitrary baseline. However, the others were modeled but not subsequently analyzed. The statistical maps of β‐coefficients were then entered into second‐level analysis. The second‐level (group) analysis was conducted using a mixed design 2(group: SCD vs. NC) × 2(condition: LS vs. LO) GLM. According to the regions involved in high‐fidelity retrieval and pathological deposition,^15^, ^16^, ^17^, ^18^, ^19^, ^20^, ^30^ results of ROIs analyses were thresholded at FWE *p* < 0.05 (small volume correction), using a CN^39^ and hippocampus and surrounding gray matter (i.e., entorhinal cortex and parahippocampal cortices) atlases^40^ as masks. Considering that the primary interest of this study was the group effect on memory retrieval, we focused on the main effect of group in the following analysis, and the main effect of condition and interaction effect (not significant) would not be displayed. Each participant's data and condition were later exported to SPSS for further analysis.

#### 2.6.3 gPPI

A mixed design 2(group: SCD vs. NC) × 2(condition: LS vs. LO) gPPI model was modeled in second‐level analysis to investigate the potential group differences and interaction effects (not significant) in FC. Cluster‐level inferences were based on nonparametric statistics from randomization/permutation analyses, with 1000 residual‐randomization iterations. Results were thresholded using a combination of a *p* < 0.01 voxel‐level threshold, and a false discovery rate (FDR) *p* < 0.05 cluster‐size threshold. In CONN, FC was represented as percent blood oxygenation level dependent (BOLD) signal change in the target ROI per unit change in BOLD signal in seed ROI. The beta values in the gPPI‐model represented the relative connectivity between seed and target ROIs during each condition compared to the gPPI‐model implicit baseline (non‐event trials). Positive values indicate increases in connectivity during one condition compared to baseline, and negative values indicate decreases in connectivity. Each participant's effect sizes of FC during high‐fidelity retrieval were later exported to SPSS for an repeated‐measures analysis of variance.

Furthermore, according to previous findings,^30^, ^31^, ^32^, ^33^ it is well established that detailed aspects of both visual input and hippocampal representations are initially monitored by the frontal lobe, while the products of retrieval are ultimately evaluated in the parietal lobe within CN. The discovered integrating region probably participate in integrating information from frontal lobe and hippocampus, subsequently transmitting the information to precuneus for post‐retrieval evaluation during high‐fidelity retrieval, forming a mediation pathway. The FCs under LS condition were thus used to conduct a mediation analysis in Mplus 8.3 (Muthén & Muthén, Los Angeles, CA, USA) to determine if the collaboration between frontal lobe and parietal lobe was mediated by the discovered integrating region.

## 3 RESULTS

### 3.1 Demographical information and neurological tests

The demographical information and performance of neurological tests of all participants are shown in Table 1. All participants showed normal scores in tests of cognition and emotion. No significant differences were found between SCD and NC groups in demographics, global cognition, and cognition in a single domain. We only found significant differences in scores of SCD‐Q9 (*t* (52) = 11.94, *p* < 0.001) and anxiety symptoms (*t* (52) = 2.99, *p* < 0.01).

**TABLE 1:** Participant demographical information and neurological tests (*N* = 54).

	NC (*n *= 28)		SCD (*n *= 26)		
Parameter	Mean (SD)	Range	Mean (SD)	Range	*p*‐value
**Demographics**					
Sex (M/F)	9/19	/	10/16	/	0.63
Age	65.71 (3.28)	61–71	66.35 (2.94)	61–72	0.46
Education (years)	12.43 (2.34)	9–18	11.77 (2.19)	9–17	0.29
**Subjective complaints and global cognition**					
SCD‐Q9	1.68 (0.84)	0–3.0	4.81 (1.08)	3.5–7.0	< 0.001
MoCA	26.61 (1.55)	25–30	26.27 (1.08)	25–28	0.36
**Episodic memory**					
PALT	9.13 (3.11)	3.5–17.5	7.87 (2.16)	4.0–11.5	0.09
AVLT immediate recall	21.32 (3.44)	13–28	20.04 (2.12)	15–24	0.11
AVLT delayed recall (5 min)	7.29 (2.19)	3–10	6.77 (1.37)	5–10	0.31
AVLT delayed recall (20 min)	7.00 (2.19)	3–10	6.38 (1.27)	4–9	0.22
**Verbal fluency**					
VF (animals)	19.75 (3.74)	13–28	20.19 (3.72)	14–30	0.67
VF (vegetables)	18.07 (4.19)	10–29	18.00 (4.14)	8–25	0.95
**Executive function**					
Digital span (forward)	8.07 (1.22)	6–11	7.54 (1.03)	6–10	0.09
Digital span (backward)	5.29 (1.27)	3–8	4.81 (1.33)	3–9	0.18
TMT A	39.96 (11.36)	20–68	42.88 (10.71)	24–67	0.34
TMT B	61.32 (15.80)	32–110	68.65 (25.73)	39–146	0.21
TMT B‐A	21.36 (14.97)	−8–61	25.77 (23.58)	−10–102	0.41
**Emotion**					
SAS	20.71 (0.90)	20–23	22.15 (2.38)	20–27	<0.01
CES‐D	0.96 (1.84)	0–7	2.12 (2.92)	0–11	0.09

*Note*: MoCA scores above 24 indicate normal global cognition; PALT scores above 3 indicate normal episodic memory; AVLT delayed recall (20 min) scores above 2 indicate normal episodic memory.

Abbreviations: AVLT, Auditory Verbal Learning Test‐Huashan; CES‐D, Center for Epidemiological Studies‐Depression; M/F, male/female; MoCA, Montreal Cognitive Assessment; NC, normal controls; PALT, Paired Associative Learning Test; SAS, Self‐rating Anxiety Scale; SCD, subjective cognitive decline; SCD‐Q9, Subjective Cognitive Decline Questionnaire 9; TMT, Trail Making Test; VF, verbal fluency test.

### 3.2 Behavioral performance

**FIGURE 2:**
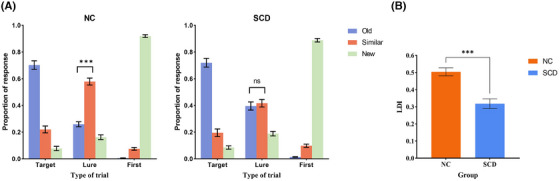
Behavioral results of MST in different groups. Panel (A): Proportion of responses for each item type. Responses to lure items differed between SCD and NC, while SCD responded fewer “similar” and more “old” to lure items than NC. Panel (B): SCD showed lower LDI compared with NC, warm color represents NC and cool color represents SCD. Error bars represent the standard error of the means. ***p < 0.001. LDI, lure discrimination index; MST, mnemonic similarity task; NC, normal controls; SCD, subjective cognitive decline.

A significant interaction of group × items × response was found [*F* (2.61, 135.89) = 9.37, *p* < 0.001, partial *η* ^2^ = 0.15]. We further found that for both groups, response main effects for lure items were significant [NC: *F* (2, 51) = 63.47, *p* < 0.001, partial *η* ^2^ = 0.71; SCD: *F* (2, 51) = 32.92, *p *< 0.001, partial *η* ^2^ = 0.56], multiple comparisons indicated a significantly higher proportion for responding “similar” to lure items than responding “old” to lure items in NC group (*p* < 0.001) but not SCD group (*p* > 0.99) (Figure 2A; Table S2). The results indicated that the proportion for responding “similar” to lure items in NC group was significantly higher than that in SCD group, while the results remained unchanged after adjusting for the similarities of lure items (Figure S1).

Besides the critical ability of correct retrieval, LDIs between the two groups were also compared (Figure 2B). The results indicated a better memory precision of NC than SCD group (*t* (52) = 5.12, *p* < 0.001). Altogether, NC population showed better accuracy in distinguishing very similar pictures and the performance remained stable after controlling false alarm rates.

### 3.3 fMRI activity during retrieval

**FIGURE 3:**
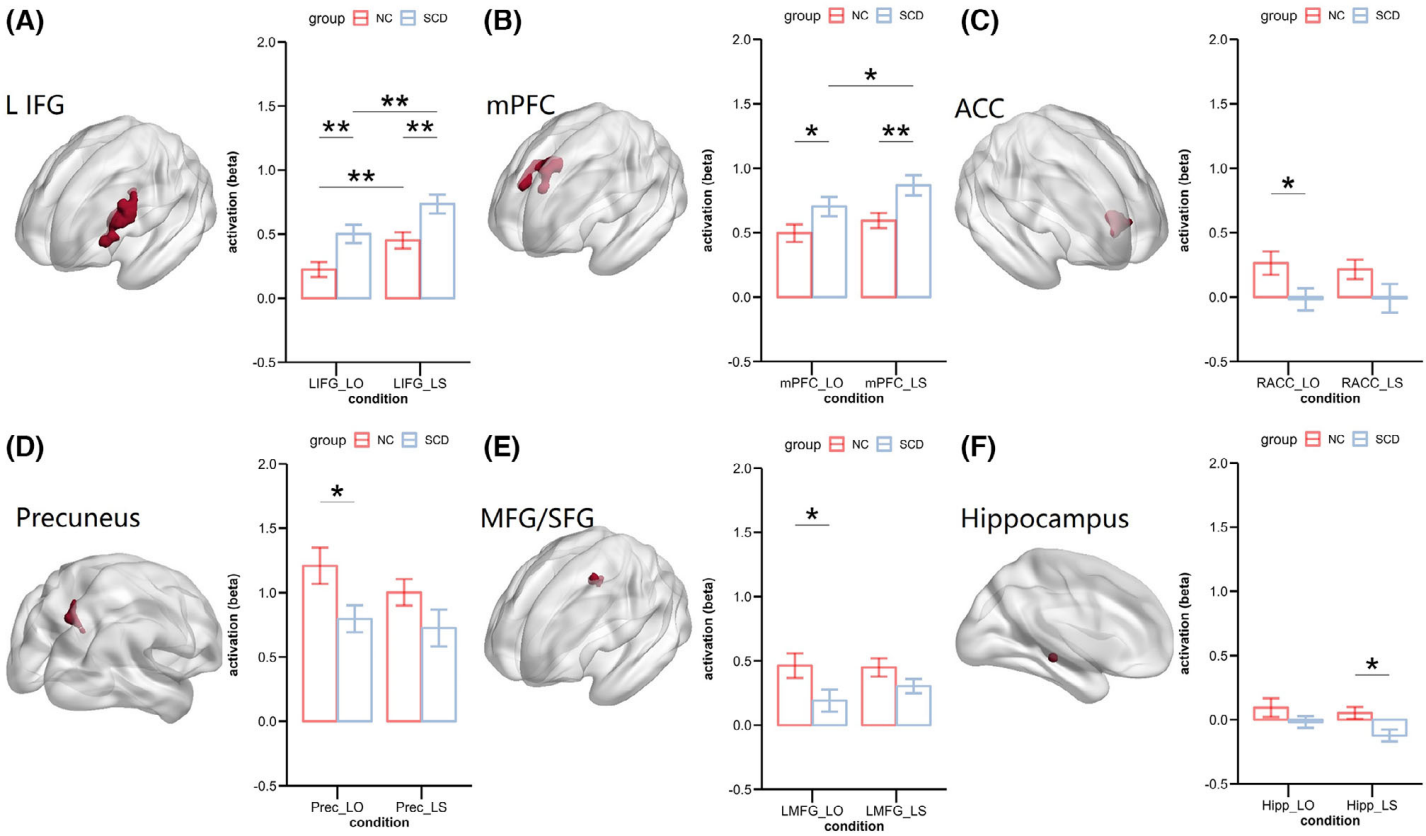
Results of 2(group: NC/SCD) × 2(condition: LS/LO) Repeated measures ANOVA in all ROIs showing significant group main effect. Panel (A)–(F): the beta value of activation under LS (high‐fidelity retrieval) and LO (low‐fidelity retrieval) conditions in ROIs showing group main effect, including the IFG, mPFC, ACC, precuneus, MFG/SFG, and the hippocampus. Red represents NC, blue represents SCD. Activation represents the beta coefficients of LS/LO in GLM compared with the baseline. Error bars represent the standard error of the means. *p < 0.05; **p < 0.01. ACC, anterior cingulate gyrus; ANOVA, analysis of variance; IFG, inferior frontal gyrus; L, left; LO, response old to lure; LS, response similar to lure; MFG/SFG, middle/superior frontal gyrus; mPFC, medial prefrontal cortex; NC, normal controls; PreC, precuneus; R, right; ROI, region of interest; SCD, subjective cognitive decline.

Significant main effects of group were listed below (Table 2). Post‐hoc analysis revealed that, under both LO and LS conditions, SCD showed higher activation than NC in IFG and mPFC (*ps* < 0.05), while the NC group showed higher activation than SCD in ACC, PreC, and MFG under the LO condition (*ps* < 0.05). In the left hippocampus, NC showed higher activation than SCD under LS (*p* = 0.01) condition. the main effect of group indicated significant differences between the two groups during the retrieval process in the aforementioned regions, particularly in the hippocampus (Figure 3A–F).

**TABLE 2:** Significant ROIs after small volume correction with masks

Brain areas	L/R	MNI coordinates peak			*p*‐value (FWE)	Cluster size (voxels)
		X	y	z		
**Control network group main effect**						
IFG	L	−44	34	8	<0.001	591
mPFC	L/R	−8	42	30	<0.001	281
ACC	L/R	4	44	12	<0.001	185
PreC	R	10	−72	46	0.008	114
MFG/SFG	L	−24	−8	48	0.027	83
**Hippocampus group main effect**						
Hippocampus	L	−32	−28	−8	0.045	12

Abbreviations: ACC, anterior cingulate gyrus; FWE, family‐wise error; IFG, inferior frontal gyrus; L/R, left/right hemisphere; MFG/SFG, middle/superior frontal gyrus; MNI, Montreal Neurological Institute; mPFC, medial prefrontal cortex; NC, normal controls; PreC, precuneus, ROI, region of interest; SCD, subjective cognitive decline.

A correlational analysis was also conducted to verify the potential link between the participants' LDIs and their brain activation during retrieval. We found a positive correlation between LDI and hippocampus activation (*r* = 0.28, *p* = 0.04) and negative correlations between LDI and activation of L IFG (*r =* −0.40, *p* < 0.01), mPFC (*r =* −0.32, *p =* 0.02) under the LS condition. Furthermore, after adjusting for demographical information (i.e., sex, age, education), correlations between LDIs and hippocampus (*r* = 0.34, *p* = 0.02), L IFG (*r* = −0.43, *p* ≤ 0.01) and mPFC (*r* = −0.31, *p* = 0.03) remained significant. The results demonstrated probable relationships between worse performance in MST and hypoactivation in the hippocampus and hyperactivation in IFG and mPFC (Table S3; Figure S2).

### 3.4 FC during retrieval

**FIGURE 4:**
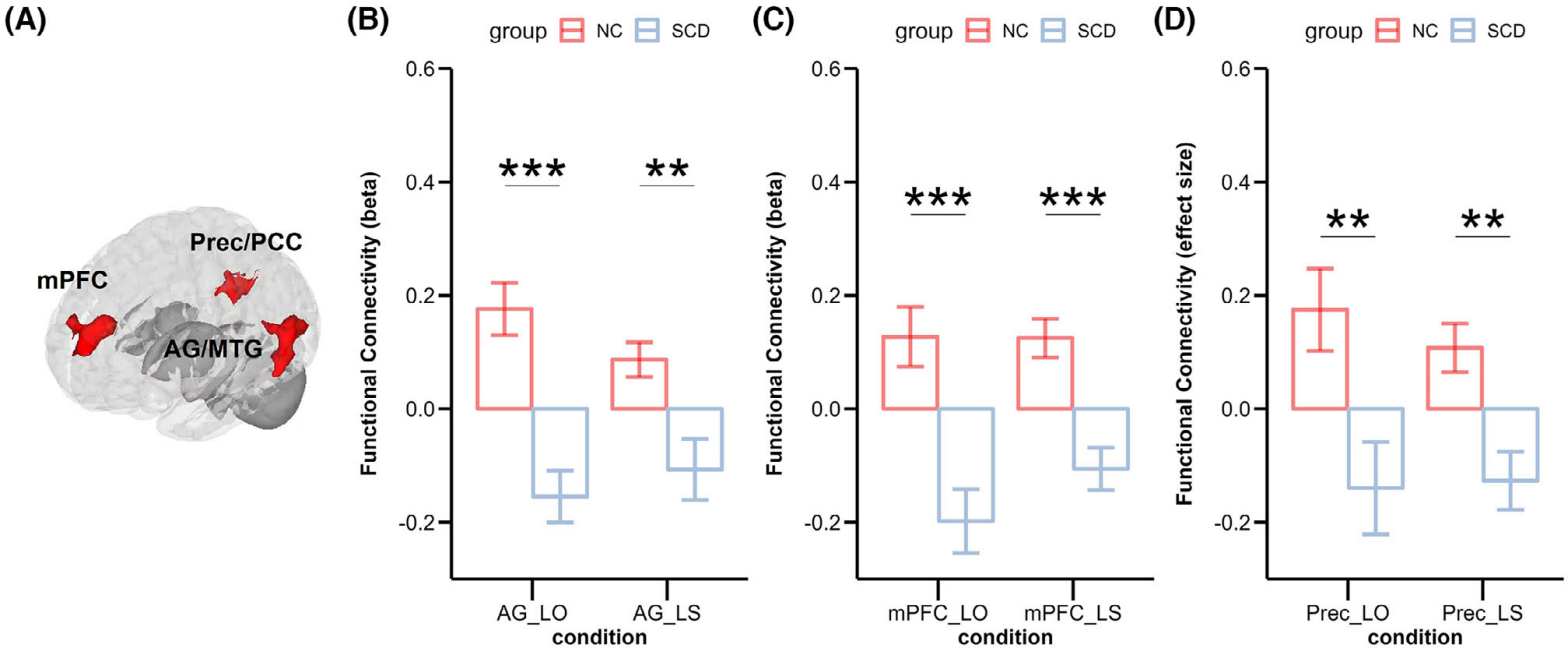
Regions showing significant group main effect in functional connectivity with the left hippocampus. Positive values mean the connectivity increases, while negative values mean the connectivity breaks down during this condition relative to trials with no events happening. Red represents NC, blue represents SCD. Panel (A): location of ROIs; Panel (B): AG/MTG cluster; Panel (C) mPFC cluster; Panel (D): PreC/PCC cluster. Error bars represent the standard error of the means. **p < 0.01; ***p < 0.001. AG/MTG, angular gyrus/middle temporal gyrus; LO, response old to lure; LS, response similar to lure; mPFC, medial prefrontal cortex; NC, normal controls; PreC/PCC, precuneus/posterior cingulate cortex; ROI, region of interest; SCD, subjective cognitive decline.

Only FC using the left hippocampus as the seed showed a significant main effect of group. Specifically, in angular gyrus/middle temporal gyrus (AG/MTG) cluster (peak MNI: −56, −58, 16; cluster size 1022 voxels, *p*‐FDR < 0.01), mPFC cluster (peak MNI: −6, 52, 28; cluster size 902 voxels, *p*‐FDR < 0.01) and left precuneus/posterior cingulate cortex (PreC/PCC) cluster (peak MNI: −6, −50, 24; cluster size 423 voxels, *p*‐FDR = 0.04), NC showed significantly higher FC with hippocampus than SCD under both conditions, *ps* < 0.01 (Figure 4). The positive betas in NC and negative betas in SCD indicated that the FCs of NC increased, while the FCs of SCD decreased during retrieval compared to baseline (non‐event trials). Notably, the AG/MTG cluster covering part of the temporal–parietal–occipital lobes was a large region consisting of the middle temporal gyrus (MTG), lateral occipital cortex, and AG. No significant effect of interaction was found.

A correlational analysis was also conducted to study the relationship between LDI and FC under LS condition. Results showed that LDI positively correlated with FC between hippocampus and mPFC cluster (*r* = 0.35, *p* = 0.01), AG/MTG cluster (*r* = 0.30, *p* = 0.03) and PreC/PCC cluster (*r* = 0.39, *p* < 0.01). The correlations remained significant (mPFC: *r *= 0.36, *p* ≤ 0.01; AG/MTG: *r *= 0.30, *p *= 0.04; PreC/PCC: *r *= 0.37, *p* ≤ 0.01) after adjusting for demographical information. The results implied possible relationships between worse LDI and lower FC between the left hippocampus and mPFC, AG/MTG, and PreC/PCC.

### 3.5 The integrating and transmitting role of AG/MTG cluster during high‐fidelity retrieval

**FIGURE 5:**
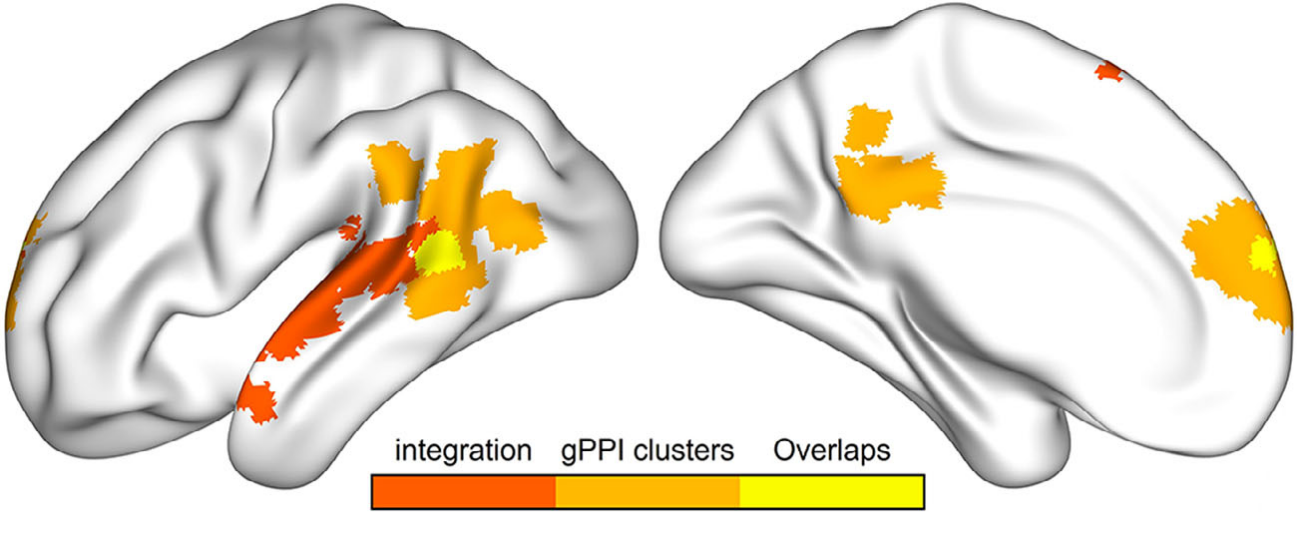
Overlaps of gPPI clusters in the current study and meta‐analysis of brain regions revolved around integration function. Common activation across all these studies was only revealed in the prefrontal cortex and AG located in the left hemisphere. Meta‐analysis map is thresholded with a false discovery rate approach with an expected FDR of 0.01. Colors from dark to light separately represent integrating regions, gPPI clusters, and overlaps. AG, angular gyrus; FDR, false discovery rate; gPPI, generalized psychophysiological interactions.

To determine which area is the possible integration and transmission region, Neurosynth (www.neurosynth.org)^41^ was used to perform an automated meta‐analysis of 771 fMRI studies using the term “integration”, and we found that the overlaps between the FC map in our results and regions only located in the mPFC cluster and AG/MTG cluster, indicating that these two regions could be the possible integration and transmission regions (Figure 5). The overlap was recomputed after adding another meta‐analysis map of term “retrieval” and only the cluster located in left AG/MTG survived, which was then set as the integrating region during retrieval to be validated (Figure S3).

In the current mediation analysis under LS condition, the monitoring signals of high‐resolution details and evaluation of products after high‐fidelity retrieval were separately represented by the beta value of FC between the hippocampus and mPFC cluster (FC_mPFC_), and FC between the hippocampus and PreC/PCC cluster (FC_PreC/PCC_). The strength of information integration and transmission was measured by FC between the hippocampus and AG/MTG cluster (FC_AG/MTG_). First, We found that correlations among FC_PreC/PCC_, FC_AG/MTG_ and FC_mPFC_ were all positively significant (*ps* < 0.001), and all of the associations remained significant after adjusting for demographical information including age, sex and education (*ps* < 0.001, partial correlation; Table S4). By conducting multiple regression, we also observed that FC_mPFC_ could significantly predict FC_AG/MTG_ (standardized *β  *= 0.73, *p* < 0.001) meanwhile both FC_mPFC_ and FC_AG/MTG_ could significantly predict FC_PreC/PCC_ (mPFC: standardized *β *= 0.35, *p* = 0.03; AG/MTG: standardized *β* = 0.31, *p* = 0.05).

**FIGURE 6:**
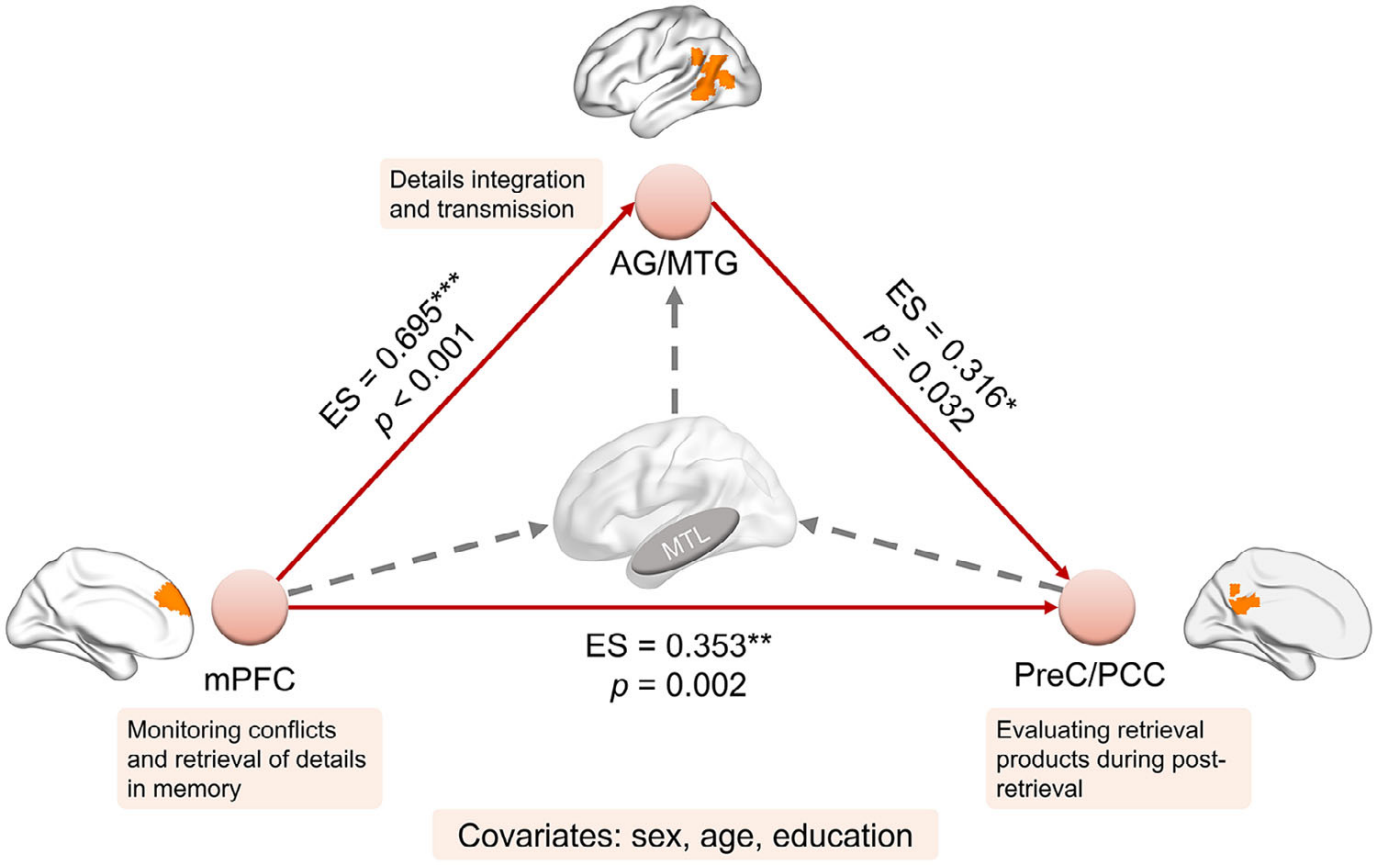
The results of mediation analysis on the mPFC‐AG/MTG‐PreC/PCC model. This model exhibited a significant indirect effect (38.22%) and a significant direct effect, supporting that the disruption to information integration and transmission pathway may affect regions' collaboration during high‐fidelity retrieval. ES means the standardized effect size. *p < 0.05 **p < 0.01; ***p < 0.001. AG/MTG, angular gyrus/middle temporal gyrus; ES: standardized effect size; FC, functional connectivity; LS, response similar to lure; mPFC, medial prefrontal cortex; PreC/PCC, precuneus/posterior cingulate cortex.

Indeed, we revealed a significant mediation effect (Figure 6). The model indicated that FC_AG/MTG_ significantly mediates the predictive effect of FC_mPFC_ on FC_PreC/PCC_ (standardized indirect effect size = 0.22, *p* = 0.04; standardized direct effect size = 0.35, *p* < 0.01; standardized total effect size = 0.57, *p* < 0.001) after adjusting for demographical information. Another mediation model of AG/MTG‐mPFC‐PreC/PCC was conducted to check whether mPFC could be the integration and transmission region but the fitness of model was worse and effect sizes were smaller than the current one (Table S5). The findings suggested that during retrieval, the synchronization of left hippocampus‐mPFC would enhance to monitor conflicts between representation and input meanwhile enhanced hippocampus‐AG/MTG synchronization serves to integrate the memory signals and monitoring signals. Ultimately, the PreC/PCC would evaluate retrieval products during post‐retrieval based on aforementioned information. In SCD individuals, this integration pathway and global brain collaboration would be disrupted, leading to negative FC, affecting high‐fidelity retrieval.

## 4 DISCUSSION

The current study demonstrates that SCD subjects have an objective cognitive decline in mnemonic discrimination compared to the NC group. This decline is related to abnormal alterations in hippocampal and CN activation, consistent with the regions involved in high‐fidelity retrieval. Additionally, it correlates with reduced FC between the hippocampus and cortical regions in the CN, such as the mPFC and PreC/PCC, as well as reduced FC between the hippocampus and the integrating region AG/MTG. Moreover, the present study reveals that dysfunction in the AG/MTG disrupts information transmission and integration, which partially contributes to the disconnection between the CN and MTL.

One major finding of the present study was that SCD is not merely subjective. Although the SCD and NC participants performed comparably on standardized neuropsychological tests and simple recognition tasks (recognition of targets), those with SCD tended to misidentify lure items as old targets, resulting in a significant decline in the LDI. These findings suggest that, while low‐fidelity memory retrieval remains intact in individuals with SCD, high‐fidelity memory retrieval is already impaired due to underlying neural disruptions. Furthermore, consistent with prior research on apolipoprotein E (APOE) carriers^26^ and mild cognitive impairment patients,^21^, ^23^ our findings indicate that subtle yet objective deficits in high‐fidelity retrieval emerge in the early stages of potential cognitive impairments. These results provide valuable supplementary evidence to prior research and demonstrate that high‐fidelity retrieval objectively declines with the progression of cognitive impairments.

In the current study, we observed dysfunctions of the hippocampus, which may underlie the mechanisms for declined high‐fidelity memory retrieval in SCD. According to the whole brain high‐fidelity retrieval model,^30^ MTL plays a central role responsible for extracting detailed memory representations, particularly distinguishing between similar items. Consistent with previous findings,^42^ our study demonstrated that SCD exhibited reduced activation in the left hippocampus compared to the NC group during high‐fidelity retrieval tasks, which correlated with poorer LDI scores. This suggests that the hippocampal dysfunction, potentially caused by pathological deposition^16^, ^17^, ^18^, ^19^, ^20^ in SCD individuals, may lead to insufficient hippocampal activation during high‐fidelity retrieval, thereby contributing to the observed decline in this capacity.

Abnormal alterations in the activation of CN were also evident in SCD. The present research found that the IFG and mPFC exhibited hyperactivation during retrieval tasks in this population, consistent with existing literature reporting heightened activity in the prefrontal cortex during retrieval among individuals with SCD or those at a higher risk for cognitive impairment.^42^ Our findings further revealed that the activation of both regions was significantly associated with poorer LDI. Previous research has posited that mPFC is involved in monitoring the retrieval process, regulating attention, and resolving potential conflicts, ensuring that relevant information is prioritized. Meanwhile, the IFG would aid in suppressing interference and monitoring the retrieval process to ensure accurate information is accessed.^25^, ^43^, ^44^, ^45^, ^46^, ^47^, ^48^, ^49^ Thus, the dysfunctions observed in the IFG and mPFC are essential for achieving high‐fidelity retrieval and the abnormal deposition in both regions^16^, ^17^, ^18^, ^19^, ^20^ might introduce noises^24^ during conflict monitoring, ultimately contributing to the decline in retrieval precision.

Furthermore, the most critical novelty of our findings is the validation of activation results through task‐related FC analyses, revealing impairments in previously unrecognized regions critical for integration and information transmission. Previous research has indicated that CN exhibits covariance in activity with the MTL during high‐fidelity retrieval in younger adults.^29^ Aligning with the high‐fidelity retrieval model,^30^ cortical regions help to resolve interference among overlapping memories and process intricate details. This study proposed that interactions between the hippocampus and control regions were correlated with accurate discrimination of lure items; in contrast, an isolated hippocampus could only achieve low‐fidelity retrieval. Our investigation into the alteration of interaction between hippocampus and CN during retrieval tasks identified this interaction as an independent factor influencing high‐fidelity retrieval, replicating findings from studies involving younger adults. Specifically, the NC group showed stronger FC than the SCD group during high‐fidelity retrieval, and stronger FC was positively correlated with higher LDI. Recent studies have also emphasized the importance of the interactions among the hippocampus, mPFC, and PreC/PCC regions, which we examined, as crucial for effective memory retrieval.^50^, ^51^, ^52^, ^53^ Moreover, reduced FC among these regions has also been linked to cognitive decline in both normal older adults and those carrying risk genes.^54^, ^55^

In addition to impaired MTL‐CN collaboration, we elucidated the critical role of the AG/MTG cluster in integrating memory information from the MTL and signals regarding similar items generated by the mPFC. This information is subsequently transmitted to the precuneus, which evaluates the retrieved memory output, comparing it to expectations and assessing its accuracy as a final checkpoint to ensure alignment with the original experience (Figure 6). In our study, while we found no significant activation in the AG, we observed a notable correlation between the FC of the MTL‐AG/MTG and LDI. Additionally, evidence supporting the mediation effect of AG/MTG on the mPFC‐PreC/PCC collaboration was identified. Prior literature has established that the temporoparietal‐occipital junction, including the AG and MTG located in the left hemisphere with extensive projections linking the frontal lobe, hippocampus, and precuneus could serve as a major connector hub and it is crucial for integrative processes.^56^, ^57^, ^58^ Specifically, the AG and MTG cluster in the left hemisphere acts as a memory buffer, storing information until it is retrieved by the CN. Concurrently, this cluster facilitates the integration of information from episodic inputs during retrieval.^32^, ^57^, ^58^ As a region extrinsic to both the CN and MTL, the AG/MTG may exhibit no direct activation but plays a vital role in supporting MTL‐CN interactions under high‐fidelity demands,^31^ emphasizing its importance in transmitting detailed information rather than processing or evaluation.^58^ Although we found no evidence of pathological deposition in the AG/MTG region, the observed failures in high‐fidelity information integration and transmission could partially explain the positive correlation between FC_AG/MTG_ and LDI, highlighting its function as a critical node mediating the interaction between the MTL and CN during high‐fidelity retrieval.^30^

Considering all the above findings of task‐related FC, our results revealed that deficits in the interaction between the hippocampal seed in MTL and cortical regions in CN, including part of mPFC, left precuneus,^59^ and the integrating region AG/MTG^31^, ^32^, ^56^, ^57^, ^58^ within left hemisphere, contribute to impaired high‐fidelity retrieval performance (LDI) in SCD. This suggests a direct relevance between the objective damage caused by cognitive impairments at an early stage and the decline in high‐fidelity retrieval abilities. While previous researchers have identified associations between resting‐state FC between the MTL and cortical regions and SCD scores, they have struggled to provide a theoretical framework for these associations due to the exploratory nature of data‐driven studies.^7^, ^24^ In contrast, our study offers compelling evidence that impairment in MTL‐CN connectivity leads to SCD symptoms—particularly in the memory domain—through gPPI analysis, and highlights potential objective impairments in high‐fidelity information integration in SCD using a sensitive paradigm that demands high‐level integration.

Overall, findings based on the regions recruited in high‐fidelity retrieval indicate that in SCD, hippocampal dysfunction leads to poor retrieval of details. Concurrent hyperactivation of the mPFC/IFG indicates low neural efficiency when monitoring and correcting errors. Consequently, the AG fails to integrate fragmented details, resulting in incomplete memory retrieval, which hinders evaluation in the precuneus due to insufficient input from the AG. The results illustrate a neural dysfunction driven by pathology rather than compensatory mechanisms. As a cognitive neuroscience model‐guided study, the findings of this study on the mechanisms of task‐related FC provide valuable insights that complement previous research utilizing resting‐state FC, which could not directly interpret the relationship between connectivity strength and cognitive performance.

In our research, we also observed that the activation of ACC and precuneus in right hemisphere was significantly lower in SCD compared to the NC group, particularly during low‐fidelity retrieval tasks (e.g., responding “old” to lure items). However, they showed no correlation with LDI, suggesting that regions in the contralateral hemisphere may play a role in attention guidance or general cognitive control, but are not primary determinants of the decline in retrieval performance within the SCD population.

In summary, this study substantiates the objective cognitive decline in the SCD population compared to cognitively normal elderly individuals and reveals the whole‐brain neural mechanisms associated with SCD contributing to deficits in high‐fidelity retrieval. Importantly, our findings highlight the failure of the AG's integrative role, thereby addressing a significant gap in previous research that predominantly emphasized the roles of the hippocampus and CN. Based on data from Chinese participants, this study advances our understanding of a critical bottleneck in the retrieval process for SCD patients and identifies a promising target for future clinical research and potential interventions, although our conclusions remain preliminary. Future investigations should employ longitudinal tracking to assess whether high‐fidelity retrieval deficits can serve as a predictive marker for the progression of SCD to cognitive impairment. Lastly, we advocate for confirmatory studies to validate the pathological mechanisms identified in the Alzheimer's disease continuum, which our research pioneers within the context of SCD.

## CONFLICT OF INTEREST STATEMENT

The authors declare no conflicts of interest.

## CONSENT STATEMENT

The study was approved by the Institutional Review Board of the Institute of Psychology, Chinese Academy of Sciences (H23104, protocol IPCAS2023014). All human subjects provided informed consent.

## Supporting information



Supporting Information

Supporting Information
