# Supplementary material for: Disrupted brain networks underlying high‐fidelity memory retrieval in subjective cognitive decline: A task‐based fMRI study
Source: Alzheimers Dement. 2024 Dec 28;21(2):e14431. doi: 10.1002/alz.14431 (PMC11848185; doi:10.1002/alz.14431)
Supplement: Supplementary file 1 — Supporting Information [file ALZ-21-e14431-s002.docx]

**Summary of the content:**

1. Supplementary figures
2. Supplementary tables

**I. supplementary figures**


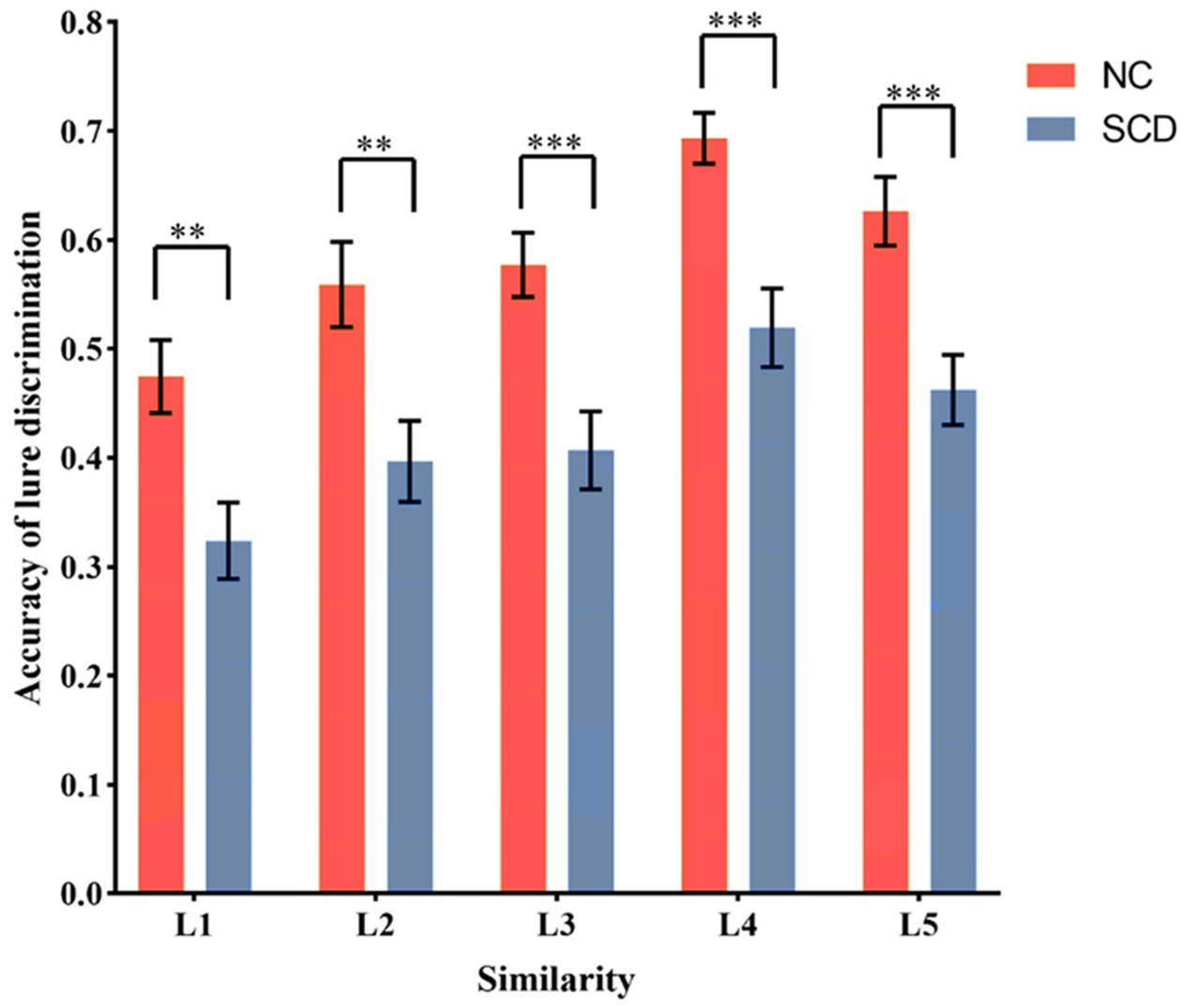


**Supplementary Figure S1.** Proportion of LS under different similarities. Proportion of LS differed between two groups under all similarities [*F*(4, 208) = 24.19, *p* < .001, partial *η*^2^ = 0.32], while no significant effects of similarities or interaction was found, indicating a significantly better performance in discriminating Lure items for NC than SCD. Error bars represent standard error of the means. **: *p* < .01; ***: *p* < .001


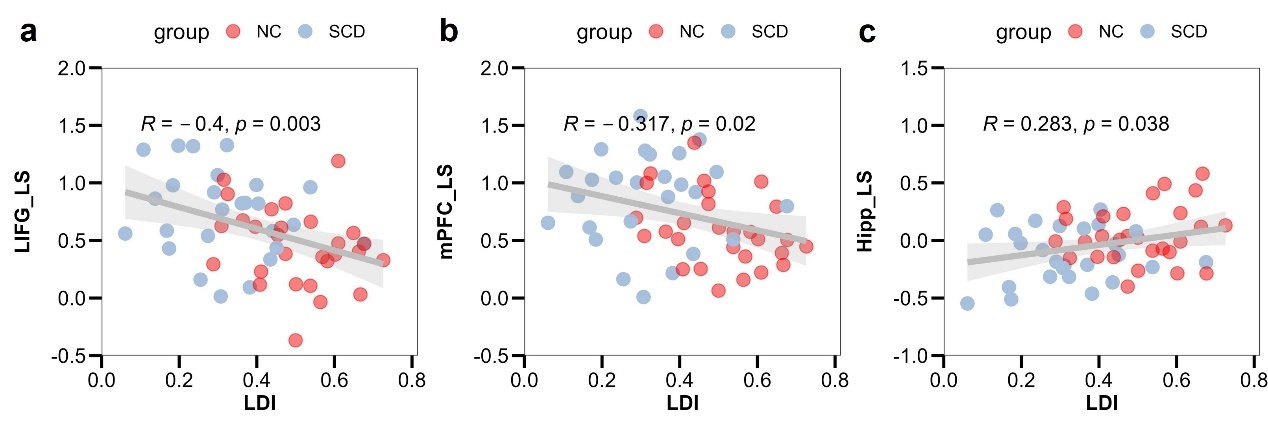


**Supplementary Figure S2.** Correlations between LDIs and IFG, mPFC and hippocampus activation under LS condition. Significant correlations between LDIs and activation in ROIs under LS condition without adjusting for covariates. Panel **a-c**: L IFG, mPFC and left hippocampus. Shaded area: 95% CI.


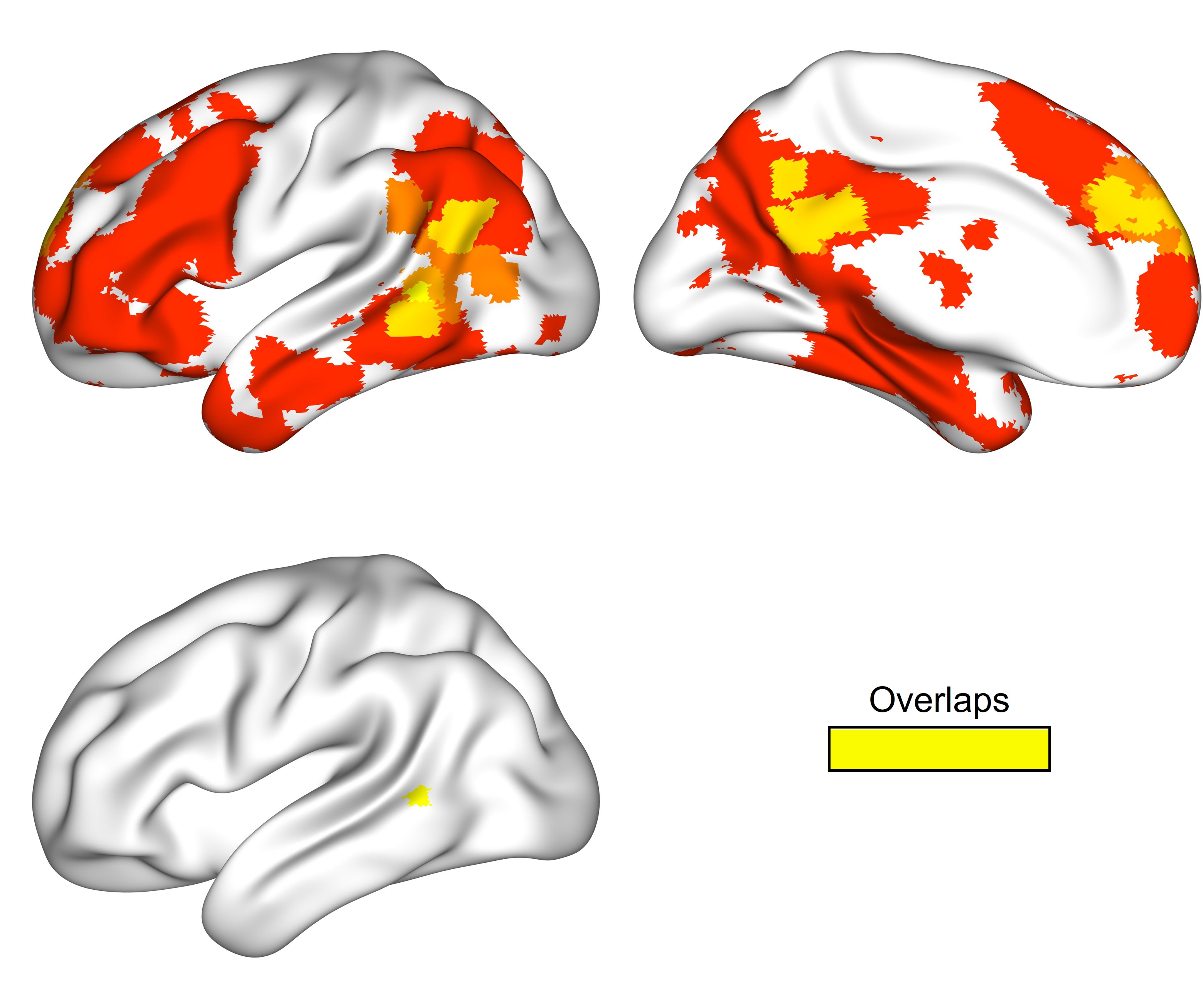


**Supplementary Figure S3.** Overlaps of gPPI clusters in the current study and Meta-analysis of brain regions of terms “integration” and “retrieval”. Common activation across all these studies were only revealed in AG/MTG cluster located in left hemisphere. Meta-analysis Map is thresholded with a false discovery rate approach with an expected FDR of 0.01. Light yellow represents the survived overlap region. AG, Angular gyrus.

**II. supplementary tables**

**Supplementary Table S1.** All codes of the 13 events defined in fMRI analysis according to participants’ responses to items. “s” represented subsequent items. Among these events, only FN (responded “new” to foil items), LO (responded “old” to lure items) and LS (responded “similar” to lure items) were later analyzed.

| Condition codes | Description |
| --- | --- |
| 1 (FN) | Responding “new” to Foil |
| 2 (FO/FS) | Responding “old/similar” to Foil |
| 3 (sTO) | Subsequently responding “old” to Target |
| 4 (TO) | Responding “old” to Target |
| 5 (sTN/sTS) | Subsequently responding “new/similar” to Target |
| 6 (TN/TS) | Responding “new/similar” to Target |
| 7 (sLS) | Subsequently responding “similar” to Lure |
| 8 (LS) | Responding “similar” to Lure |
| 9 (sLO) | Subsequently responding “old” to Lure |
| 10 (LO) | Responding “old” to Lure |
| 11 (sLN) | Subsequently responding “new” to Lure |
| 12 (LN) | Responding “new” to Lure |
| 13 | Others (omissions, misses…) |

**Supplementary Table S2.** Proportions of All participants responses to different item types

|  | NC (*n*=28) | | SCD (*n*=26) | |
| --- | --- | --- | --- | --- |
| Propotion | Mean (SD) | range | Mean (SD) | range |
| **old\|Target** | 0.703 (0.169) | 0.345-0.979 | 0.720 (0.165) | 0.380-0.947 |
| similar\|Target | 0.220 (0.136) | 0.010-0.522 | 0.195 (0.143) | 0.021-0.573 |
| new\|Target | 0.077 (0.085) | 0.000-0.337 | 0.085 (0.064) | 0.021-0.242 |
| **old\|Lure** | 0.259 (0.101) | 0.104-0.483 | 0.395 (0.156) | 0.094-0.708 |
| **similar\|Lure** | 0.579 (0.138) | 0.358-0.840 | 0.416 (0.144) | 0.167-0.812 |
| new\|Lure | 0.162 (0.094) | 0.011-0.344 | 0.188 (0.087) | 0.053-0.365 |
| old\|First | 0.006 (0.009) | 0.000-0.032 | 0.014 (0.014) | 0.000-0.057 |
| similar\|First | 0.075 (0.054) | 0.014-0.236 | 0.098 (0.060) | 0.007-0.245 |
| **New\|First** | 0.920 (0.055) | 0.746-0.986 | 0.888 (0.066) | 0.734-0.982 |
| LDI | 0.504 (0.124) | 0.288-0.726 | 0.318 (0.143) | 0.061-0.676 |
| *Note:* the proportions for responses in MST. E.g., similar\|Lure represents the proportion for “responding ‘similar’ to ‘Lure’ items”. | | | | |

**Supplementary Table S3.** The correlation between LDIs and beta of brain activation under the LS condition

| ROIs | *r* | *p* | | *r*  *(adjusted)* | | *p* |
| --- | --- | --- | --- | --- | --- | --- |
| **ROIs of Group main effect** | | | | | | |
| L Hippocampus | 0.283 | .038* | 0.335 | | .016* | |
| L IFG | -0.400 | .003** | -0.426 | | .002** | |
| mPFC | -0.317 | .019* | -0.314 | | .025* | |
| ACC | 0.114 | .413 | 0.114 | | .424 | |
| R PreC | 0.224 | .103 | 0.219 | | .122 | |
| L MFG/SFG | 0.093 | .505 | 0.117 | | .414 | |
| Note: covariates included sex, age, education.  *: *p* < .05; **: *p* < .01 | | | | | | |

**Supplementary Table S4.** The correlation among betas of FC between hippocampus and all significant clusters under the LS condition in both groups

| *β* of FC | *r* | | | | partial correlation *r* | | |
| --- | --- | --- | --- | --- | --- | --- | --- |
|  | AG/MTG | PreC/PCC | mPFC | AG/MTG | | PreC/PCC | mPFC |
| AG/MTG |  |  |  |  | |  |  |
| PreC/PCC | 0.576  (*p* < .001) |  |  | 0.582  (*p* < .001) | |  |  |
| mPFC | 0.695  (*p* < .001) | 0.540  (*p* < .001) |  | 0.712  (*p* < .001) | | 0.592  (*p* < .001) |  |
| Note: covariates included sex, age, education. FC, functional connectivity; LS, response similar to Lure; AG/MTG, Angular gyrus/Middle Temporal Gyrus; PreC/PCC, precuneus/posterior cingulate cortex; mPFC, medial prefrontal cortex.  *: *p* < .05; **: *p* < .01 | | | | | | | |

**Supplementary Table S5.** The comparison between two possible mediation models.

| Models | mPFC→AG/MTG→PreC/PCC | AG/MTG→ mPFC→PreC/PCC |
| --- | --- | --- |
| AIC | -39.918 | -51.188 |
| BIC^†^ | -51.445 | -62.715 |
| Fitness | χ^2^ = 2.867, *p* =.413 | χ^2^ = 4.861, *p* =.182 |
| RMSEA | *p* = .472 | *p* = .233 |
| CFI/TLI | 1.000/1.006 | 0.971/0.914 |
|  | | |
| Total effect^‡^ | 0.573 (*p* < .001) | 0.547 (*p* < .001) |
| Direct effect^§^ | 0.353 (61.6%, *p* = .002) | 0.307 (56.1%, *p* = .034) |
| Indirect effect^¶^ | 0.219 (38.2% *p* = .035) | 0.239 (43.7%, *p* = .003) |
| ^†^Sample-Size Adjusted BIC  ^‡^Standardized total effect size  ^§^Standardized direct effect size  ^¶^Standardized indirect effect size  Note: Left: the current model; Right: the alternative model. The current model has a larger CFI/TLI and total effect size, suggesting a better explanation for the group data. AG/MTG, Angular gyrus/Middle Temporal Gyrus; PreC/PCC, precuneus/posterior cingulate cortex; mPFC, medial prefrontal cortex. AIC: Akaike Information Criterion; BIC: Bayesian Information Criterion; RMSEA: Root Mean Square Error of Approximation; CFI: Comparative Fit Index;TLI: Tucker-Lewis Index. | | |
